# Proanthocyanidins from *Vaccinium vitis-idaea* L. Leaves: Perspectives in Wound Healing and Designing for Topical Delivery

**DOI:** 10.3390/plants11192615

**Published:** 2022-10-05

**Authors:** Gabriele Vilkickyte, Modestas Zilius, Vilma Petrikaite, Lina Raudone

**Affiliations:** 1 Laboratory of Biopharmaceutical Research, Institute of Pharmaceutical Technologies, Lithuanian University of Health Sciences, Sukileliu Av. 13, LT-50162 Kaunas, Lithuania; 2 Laboratory of Pharmaceutical Sciences, Institute of Pharmaceutical Technologies, Lithuanian University of Health Sciences, Sukileliu Av. 13, LT-50162 Kaunas, Lithuania; 3 Department of Clinical Pharmacy, Lithuanian University of Health Sciences, Sukileliu Av. 13, LT-50162 Kaunas, Lithuania; 4 Laboratory of Drug Targets Histopathology, Institute of Cardiology, Lithuanian University of Health Sciences, Sukileliu Av. 13, LT-50162 Kaunas, Lithuania; 5 Department of Pharmacognosy, Lithuanian University of Health Sciences, Sukileliu Av. 13, LT-50162 Kaunas, Lithuania

**Keywords:** lingonberry, proanthocyanidins, polymeric films, film properties, wound healing

## Abstract

The compositions and health-beneficial properties of lingonberry leaves (*Vaccinium vitis-idaea* L.) are well established; however, their proanthocyanidins are still heavily underutilized. Optimizing their delivery systems is key to enabling their wider applications. The present study investigates the phytochemical and ‘wound-healing’ properties of proanthocyanidin-rich fraction(s) (PRF) from lingonberry leaves as well as the development of optimal dermal film as a proanthocyanidin delivery system. The obtained PRF was subjected to HPLC-PDA and DMAC analyses to confirm the qualitative and quantitative profiles of different polymerization-degree proanthocyanidins. A ‘wound healing’ in vitro assay was performed to assess the ability of PRF to modulate the wound environment for better healing. Low concentrations of lingonberry proanthocyanidins were found to accelerate ‘wound‘ closures, while high levels inhibited human fibroblast migration. Fifteen dermal films containing PRF were prepared and evaluated based on their polymer (MC, HEC, PEG 400) compositions, and physical, mechanical, and biopharmaceutical properties using an experimental design. The composition containing 0.30 g of MC, 0.05 g of HEC, and 3.0 g of PEG 400 was selected as a promising formulation for PRF delivery and a potentially effective functional wound dressing material, supporting the need for further investigations.

## 1. Introduction

Lingonberry (*Vaccinium vitis-idaea* L.), a small evergreen shrub of the family Ericaceae, is among the best natural sources of bioactive compounds with beneficial health effects [1]. This plant is particularly valued for its content of proanthocyanidins, representing up to 71% of the total phenolic compounds [2]. Proanthocyanidins, also known as condensed tannins, are colorless flavonoids composed of flavan-3-ol units. These phenolic compounds are considered safe and effective natural antioxidants. Extensive research has indicated that proanthocyanidins exhibit anti-inflammatory, immunomodulatory, anticancer, antibacterial, and hypolipidemic activities [3,4,5,6,7]. They possess systemic as well as topical action. Skin care products with proanthocyanidins have anti-UV, anti-aging, brown spot-lightening, and anti-wrinkle effects [8,9]. Recently, proanthocyanidins were found to hasten the wound contractions and closure processes [10,11]; they are seen as promising candidates for the development of new dermal forms for wound healing.

The skin’s structural integrity is disrupted via acute or chronic injuries, requiring recovery of the skin barrier function, reconstruction of tissues, and maintenance of internal homeostasis [12]. Wounds affect people of all ages and can be the major cause of reduced quality of life due to severe pain and aesthetical unacceptability [13,14,15]. The common ways to treat wounds include debridement or the removal of non-viable tissue, irrigation, topical antibiotics, tissue grafts, and autolytic or enzymatic mechanisms, which possess major drawbacks and undesirable side effects [15,16]. Since there is plenty of research into the active constituents of plants that promote (or modulate) wound healing, the utility of plant extracts is gaining more popularity and acceptability among people [17,18]. The use of plant resources alone or in combination with synthetic agents gives beneficial value to wound healing by contributing to antimicrobial activity, working as free radical scavengers in the wound site, and enhancing mitogenic activity, angiogenesis, and collagen production [16,17].

Optimizing the dosage form is critical to achieving clinical efficacy and safety. The pharmaceutical product must be designed in such a way that the biologically active substance can penetrate and enhance its effects [19,20]. A great variety of dermal forms are available for managing acute or chronic non-healing wounds. Commonly used topical formulations include ointments, creams, and gels, which are convenient to use but can be easily washed or wiped off by a patient’s clothes; therefore, a repeated application is needed. Medicinal patches attempt to solve these problems; however, they have other disadvantages, namely skin irritation, sweat duct obstruction, pain while peeling off, and a sticky feel after application [19,20,21,22]. To improve patient compliance, there is a need for the development of topical preparations. Film-forming systems were proposed as a novel alternative to conventional topical and dermal formulations [19,20,23]. Designing thin films requires in-depth knowledge of the biological and pharmaceutical properties of active substances, polymers, and plasticizers, as well as suitable production technologies [24,25].

Numerous studies about the development of pharmaceutical forms can be found in the literature, but data concerning the optimization of the performances of thin films are few [20,24]. Since natural skin care medicinal products are in demand [18] and lingonberry leaves containing high levels of proanthocyanidins are underutilized [26], an interesting solution is to incorporate lingonberry proanthocyanidins into polymeric films to provide possible wound-healing effects. No study, to our knowledge, has yielded the purification of lingonberry leaf proanthocyanidins, incorporating them into developed dermal films as active phytochemicals that modulate wound healing.

Our research evaluated the phytochemical profiles and ‘wound-healing’ properties of lingonberry’s proanthocyanidin-rich fraction (PRF), selected the optimal compositions, and determined the physical and mechanical properties of methyl-cellulose-hydroxyethyl cellulose films containing PRF.

## 2. Results

### 2.1. Phytochemical Profiling of PRF

**Figure 1:**
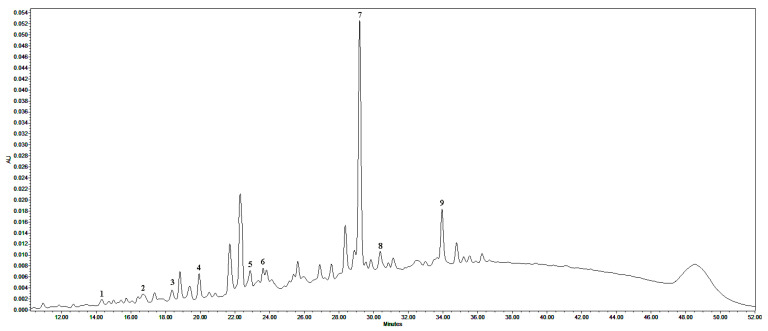
Representative HPLC-PDA chromatogram of PRF at 280 nm. Peak assignments: 1—procyanidin B1, 2—procyanidin B2, 3—(+)-catechin, 4—procyanidin B3, 5—(−)-epicatechin, 6—procyanidin C1, 7—procyanidin A1, 8—procyanidin A4, and 9—procyanidin A2.

A representative HPLC-PDA chromatogram of PRF is presented in Figure 1. Monomeric flavan-3-ols ((+)-catechin, (−)-epicatechin), A-type dimeric (procyanidin A1, A2, and A4), B-type dimeric (procyanidins B1, B2, and B3), and B-type trimeric (procyanidin C1) proanthocyanidins were identified in a tested fraction of lingonberry leaves. The sum of the identified compounds reached 98.9 ± 0.6 mg/g dry weight (DW). Procyanidin A1 was the principal compound (50.6 ± 0.3 mg/g DW) followed by procyanidin A2 (16.5 ± 0.5 mg/g DW), accounting for 51.1% and 16.7%, respectively, of the total identified proanthocyanidins. The maximum absorption of other peaks was around 280 nm, suggesting that unidentified compounds have tannin origins, while the baseline (with an upward drift) along with the retention time indicated a possible low resolution of high polymerization-degree proanthocyanidins. The DMAC method confirmed a 3.8-fold higher level of proanthocyanidins in PRF as the total amount of 376.7 ± 2.9 mg/g DW was calculated.

### 2.2. ‘Wound-Healing’ Properties of PRF

**Figure 2:**
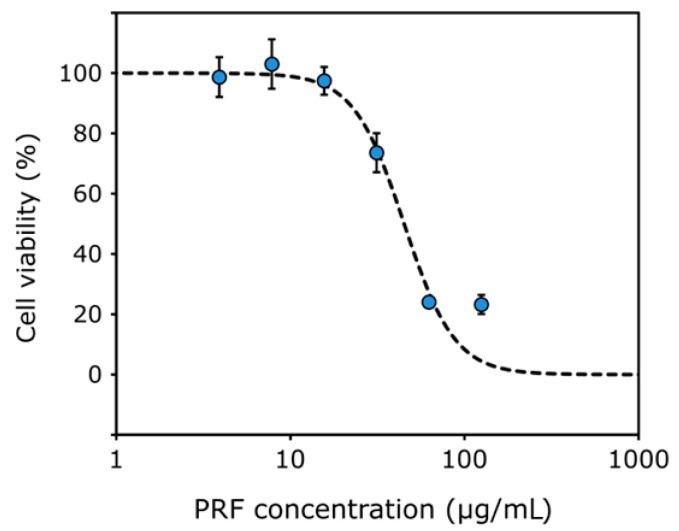
PRF effect on HF cell viability after 72 h of incubation by MTT assay. Datapoints are experimental values (averages of at least two repeats) while the line is a fit of the standard inhibition model with the Hill coefficient of 3.

The additional cytotoxicity experiment by the MTT assay was performed on human foreskin fibroblast (HF) to evaluate the PRF effect on HF viability in the range of concentrations from 3.9 to 125 µg/mL (Figure 2). It was found that PRF at a concentration of 20 µg/mL reduces HF viability up to ~10% even after 72 h of incubation. Thus, the RPF effect on HF migration was tested at 20, 10, and 5 µg/mL concentrations, with expectations not to significantly reduce the cell proliferation.

**Figure 3:**
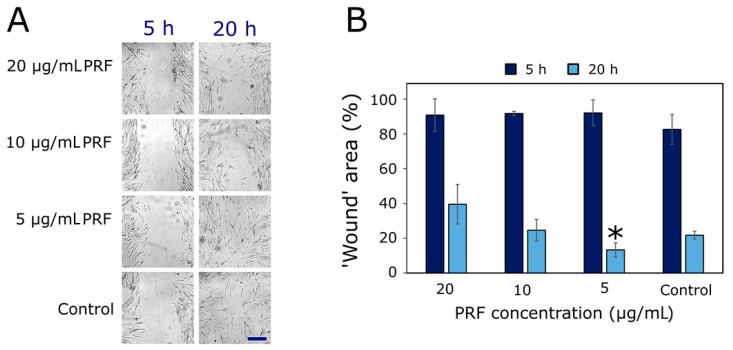
PRF effect on human fibroblast migration. (**A**) Photos of the ‘wound’ area in the human fibroblast monolayer after 5 and 20 h of incubation with 20, 10, and 5 µg/mL of PRF, and control (only medium). (**B**) Quantitative calculations of the ‘wound’ area (%). The scale bar indicates 100 µm. Asterisk (*) indicates *p* < 0.05, *n* = 3.

The PRF showed a concentration- and time-dependent effect on HF migration by the ‘wound healing’ assay (Figure 3). The stronger effect and smaller area of the ’wound‘are seen after a longer incubation. The highest tested concentration of 20 µg/mL most efficiently (1.8-fold) inhibited the cell migration (‘wound‘ area after 20 h was 39.5 ± 11.4%), while the lowest concentration of 5 µg/mL statistically significantly increased (1.6-fold) HF migration (the ‘wound’ area after 20 h was 13.3 ± 4.1%), compared to the control (the ‘wound’ area after 20 h was 21.8 ± 2.3%).

### 2.3. Characterization of Experimental Polymeric Films

#### 2.3.1. Thickness of Films

Quality parameters of experimental methylcellulose-hydroxyethyl cellulose (MC-HEC) films are given in Table 1. The thickness of the experimental polymeric films was in the range of 262–522 µm. The MH0.350-P2.0 film (No. 13) had the lowest thickness (262 ± 18 µm). This film did not differ statistically significantly (*p* ≥ 0.05) from MH0.400-P2.0 (No. 6) and MH0.400-P2.0 (No. 10) films with thicknesses of 285 ± 13 µm and 304 ± 6 µm, respectively. The MH0.425-P2.5 film (No. 9) had the highest thickness (522 ± 33 µm). This film differed statistically significantly (*p* < 0.05) from other experimental MC-HEC films. It appeared that, as the amount of PEG 400 in the film increased, its thickness increased. This was confirmed by a statistically significant (*p* < 0.05) moderate correlation (*r* = 0.624) between the amount of PEG 400 and film thickness.

#### 2.3.2. Moisture of Films

The moisture content in the experimental MC-HEC films ranged from 3.9 to 7.1% (Table 1). The lowest moisture content (3.9 ± 0.3%) was in the experimental polymeric film (MH0.450-P3.0, No. 4) containing 0.35 g of methylcellulose, 0.10 g of hydroxyethyl cellulose, and 3.0 g of PEG 400. The highest moisture content (7.1 ± 1.0%) was in the polymeric film (MH0.400-P2.0, No. 6) containing 0.35 g of methylcellulose, 0.05 g of hydroxyethyl cellulose, and 2.0 g of PEG 400. There was no statistically significant difference (*p* ≥ 0.05) among:

(a) Experimental MC-HEC films (Nos. 1–5, 7, 9, 14, 15) contained 2.5–3.0 g of PEG 400, and the moisture content in the film ranged from 3.9 to 5.1%;
(b) Experimental MC-HEC films (Nos. 6, 10–13) contained 2.0 g of PEG 400 and MH0.375-P2.5 film (No. 8) contained 2.5 PEG 400; the moisture content of the films ranged from 5.5 to 7.1%.

**Figure 4:**
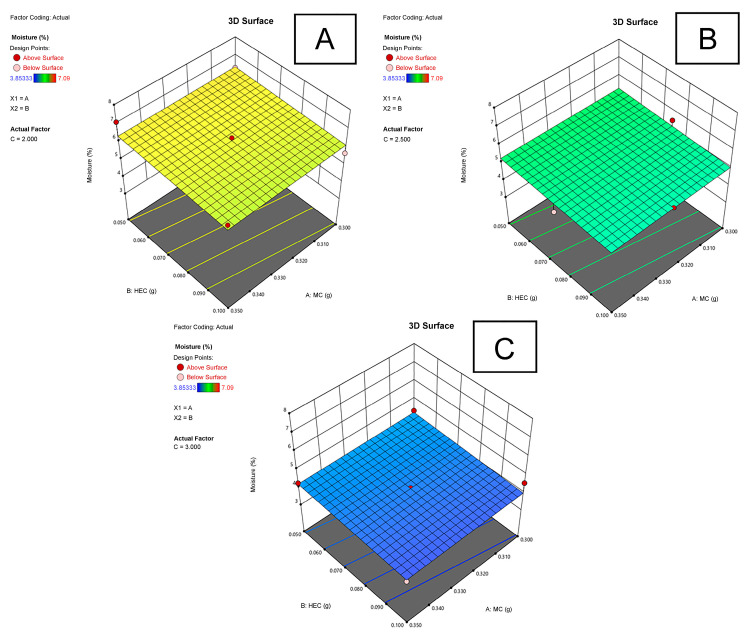
The dependence of the moisture (%) of the experimental films on the amount (g) of MC and HEC as the amount (g) of PEG 400 in the film is (**A**) 2.0 g, (**B**) 2.5 g, (**C**) 3.0 g.

It was found that as the amount of PEG 400 in the film increased, the moisture content in it decreased (Figure 4). This was confirmed by a statistically significant (*p* < 0.01) and very strong correlation (*r* = −0.918) between the amount of PEG 400 and the moisture content.

#### 2.3.3. Stickiness of Films

The stickiness of the experimental MC-HEC films ranged from 0.143 to 0.419 N (Table 1). The MH0.400-P2.0 film (No. 6), containing 0.35 g of methylcellulose, 0.05 g of hydroxyethyl cellulose, and 2.0 g of PEG 400, had the lowest stickiness (0.143 ± 0.017 N). This film did not differ statistically significantly (*p* ≥ 0.05) from MH0.450-P2.0 (No. 12) film, containing 0.35 g of methylcellulose, 0.10 g of hydroxyethyl cellulose, and 2.0 g of PEG 400, with a stickiness of 0.179 ± 0.017 N.

**Figure 5:**
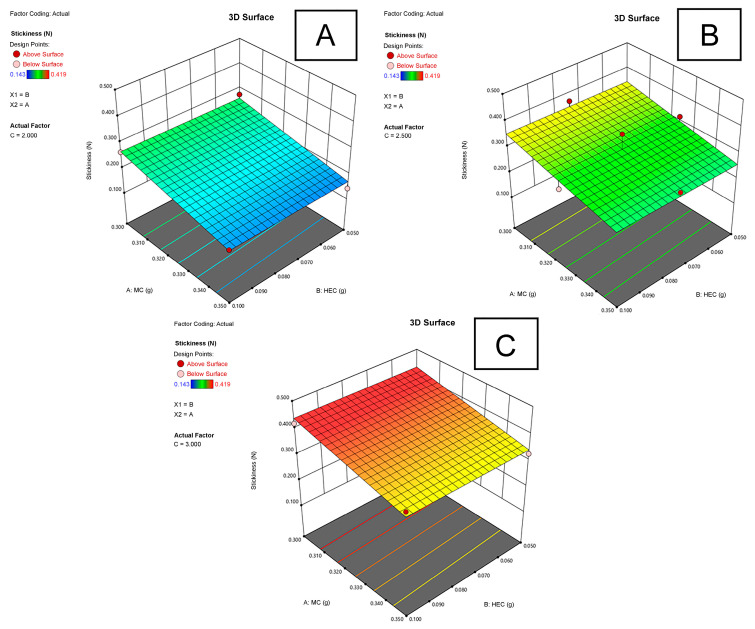
The dependence of the stickiness (N) of the experimental films on the amount (g) of MC and HEC as the amount (g) of PEG 400 in the film is (**A**) 2.0 g, (**B**) 2.5 g, (**C**) 3.0 g.

The MH0.400-P3.0 film (No. 3), containing 0.30 g of methylcellulose, 0.10 g of hydroxyethyl cellulose, and 3.0 g of PEG 400, had the highest stickiness (0.419 ± 0.012 N). This film did not differ statistically significantly (*p* ≥ 0.05) from MH0.350-P3.0 (No. 14) and MH0.400-P3.0 (No. 5) films with a stickiness of 0.412 ± 0.027 N and 0.386 ± 0.024 N, respectively. It was found that by increasing the amount of PEG 400 and decreasing the amount of methylcellulose in the film, its stickiness increased (Figure 5). However, only a statistically significant (*p* < 0.01) strong correlation (*r* = 0.851) was found between the amount of PEG 400 and film stickiness. Results showed that as the moisture content of the film decreased, its stickiness increased. This was confirmed by a statistically significant (*p* < 0.01) strong correlation (*r* = −0.751) between the moisture content and film stickiness.

#### 2.3.4. Proanthocyanidins Release from Films

**Figure 6:**
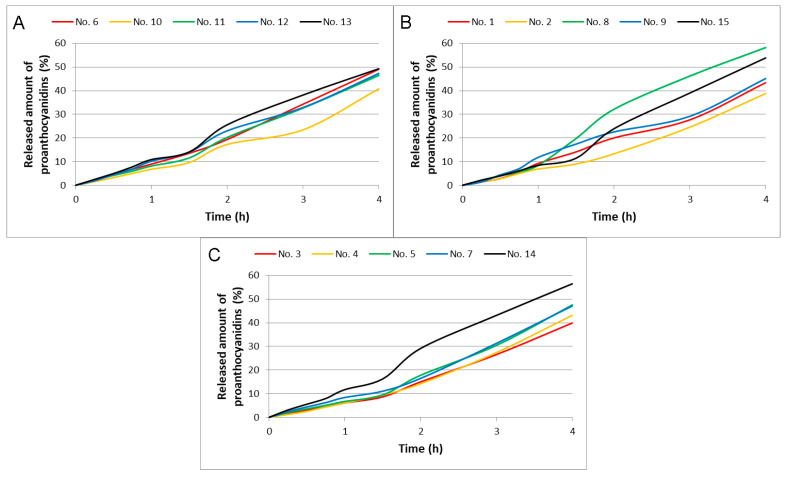
The released amount of proanthocyanidins from the experimental polymeric films as the amount (g) of PEG 400 in the film is (**A**) 2.0 g, (**B**) 2.5 g, (**C**) 3.0 g.

The release kinetics of proanthocyanidins from experimental MC-HEC films are shown in Figure 6. The release profiles of these compounds corresponded to a zero-order model (*R*^2^ = 0.9657–0.9942), which shows a linear relationship between the total released amount of proanthocyanidins (%) and the duration of the process (h).

Experimental MC-HEC films (No. 6, 10, 11, 12, 13) containing 2.0 g of PEG 400 released 40.8–49.3% (flux 188.7–233.0 µg/cm²) proanthocyanidins after 4 h (Figure 6A). Of these films, the most (49.3 and 49.1%, respectively) proanthocyanidins were released from MH0.350-P2.0 (No. 13) and MH0.400-P2.0 (No. 6) films containing 0.30–0.35 g of methylcellulose and 0.05 g of hydroxyethyl cellulose. The lowest release of these compounds (40.8%) was from MH0.400-P2.0 film (No. 10) containing 0.30 g of methylcellulose and 0.10 g of hydroxyethyl cellulose.

Experimental MC-HEC films (No. 1, 2, 8, 9, 15) containing 2.5 g of PEG 400 released 38.7–58.2% (flux 206.3–294.8 µg/cm²) proanthocyanidins after 4 h (Figure 6B). Of these films, the most (58.2 and 53.8%, respectively) proanthocyanidins were released from MH0.375-P2.5 (No. 8) and MH0.375-P2.5 (No. 15) films containing 0.300–0.325 g of methylcellulose and 0.050–0.075 g of hydroxyethyl cellulose. The lowest release of these compounds (38.7%) was from MH0.400-P2.5 film (No. 2) containing 0.325 g of methylcellulose and 0.075 g of hydroxyethyl cellulose.

Experimental MC-HEC films (No. 3, 4, 5, 7, 14) containing 3.0 g of PEG 400 released 39.8–56.5% (flux 211.4–293.7 µg/cm²) proanthocyanidins after 4 h (Figure 6C). Of these films, the most (56.5%) proanthocyanidins were released from MH0.350-P3.0 (No. 14) film containing 0.30 g of methylcellulose and 0.05 g of hydroxyethyl cellulose. The lowest release of these compounds (39.8%) was from MH0.400-P3.0 film (No. 3) containing 0.30 g of methylcellulose and 0.10 g of hydroxyethyl cellulose.

Statistical analysis showed a statistically significant (*p* < 0.01) moderate inverse correlation (*r* = −0.643) between the amount of hydroxyethyl cellulose in the films and the released amount of proanthocyanidins after 4 h.

#### 2.3.5. Selection of the Film

After the analysis of the data on thickness, moisture, stickiness, and release (0.25, 0.5, 0.75, 1, 2, 3, and 4 h), only statistically significant linear models of moisture, stickiness, and release after 0.25 h were obtained (Section 4.6.1). These quality parameters were selected as optimization criteria. According to them, the composition of the polymeric films with the highest desirability value (0.869) was obtained, which shows the percentage compliance with the set criteria (in our case it would be 86.9%): 0.30 g of MC, 0.05 g of HEC, and 3.0 g of PEG 400. Table 2 shows theoretical and experimental values of the physical, mechanical, and biopharmaceutical properties of this film.

These selected compositions of the polymeric films corresponded to composition No. 14 of the experimental design; the practical values for the appropriate properties are given in Table 3. The experimental stickiness value of the optimal composition of the MC-HEC film was 4.6% lower than the theoretical value. The experimental values of moisture and release after 0.25 h were 2.3% and 20% higher than the theoretical values, respectively.

## 3. Discussion

The interest in utilizing proanthocyanidins in the pharmaceutical industry has considerably increased over time with the discovery of their beneficial functions [27]. One possible approach is via skin application and incorporation into dermal formulations, such as polymeric films, which offer many advantages in terms of effective drug delivery and improved patient compliance [19,20]. However, to design an efficient thin film for the treatment of skin conditions, the following are needed: the purification of phytoconstituents, qualitative and quantitative analyses, biological property evaluations, and optimization of film formulations [24]. These steps were taken during the present study to model the polymeric film containing lingonberry proanthocyanidins with potential wound-healing properties.

Lingonberry fruits are highly valued in the food and nutraceuticals industry, while leaves, despite the considerable richness of proanthocyanidins and availability during all seasons, are still hardly used [28]. Due to the complexity of the matrix of lingonberry leaves, fractionation using Sephadex LH-20 was suggested, resulting in the proanthocyanidins-bounded fraction [29]. Present HPLC-PDA results showed that the PRF fraction was rich in different polymerization degree compounds: monomeric flavan-3-ols, and A- or B-type dimers and trimers. One primary problem with the analysis of proanthocyanidins is that due to the complexities of the structures and different linkages, the results of analytical procedures can often be erroneous, non-reproducible, non-selective, or not quantifiable [30]. Previously, PRF was additionally analyzed by our research group using the UPLC-PDA/ESI-QTOF-MS method to confirm the identity of proanthocyanidins and suggest more A-type procyanidin trimers [31]. Other studies indicated that oligomeric (mDP 4–10) and polymeric (mDP > 10) proanthocyanidins can be found in the lingonberry matrix as well [2,32], thus suggesting unidentified larger polymeric molecules in our tested sample. Nevertheless, liquid chromatography coupled to photodiode-array or mass detection may be useful for authentication, but it is not suitable for accurate quantification since not all analytical standards are available and response factors for the individual polymers are unknown [33,34]. Quantification problems are usually overcome by the DMAC spectrophotometric assay, which is regarded as a simple, rapid, robust, and relatively specific technique for the evaluation of the total amount of proanthocyanidins [34,35,36]. The DMAC analysis used in the present study revealed much more proanthocyanidins in the fraction of lingonberry leaves than determined with the HPLC-PDA method, showing that not all proanthocyanidins were identified. Therefore, the DMAC assay was chosen for further analysis of polymeric films containing proanthocyanidins.

The literature pertaining to the biological activities of proanthocyanidins strongly suggests possible application in wound healing [10,37]. Wound healing comprises many processes, such as hemostasis, coagulation, inflammation, proliferation, epithelialization, contraction of the wound, and others, thus involving not only recovery of skin barrier integrity, but also suppression of inflammation and secondary complications [12,17,21]. Consequently, the main effects of plant secondary metabolites working toward wound healing can be addressed as cell migration and proliferation as well as antimicrobial, anti-inflammatory, and antioxidant activities [17]. Previously, our group reported that PRF from lingonberry leaves surpassed all other phenolic fractions and crude extracts by the highest antioxidant, anti-inflammatory potential, and strongest antimicrobial properties [31]. In addition, cytotoxic PRF activity has been established in a previous experiment against human colon adenocarcinoma HT-29, renal carcinoma CaKi-1, and melanoma IGR39 cell lines, and was in the range from 30 to 50 µg/mL [29]. Thus, it was expected that PRF could also affect the viability of human fibroblasts, which serve pivotal roles in extracellular matrix reorganization during wound contractions. Fibroblast migration to (and proliferation within) the wound sites is critical for granulation and the end state of the wound [38,39].

A PRF fraction at 20 µg/mL and lower concentrations did not significantly reduce the viability of HF, indicating that proanthocyanidins do not decrease fibroblast proliferation and functions and do not delay epithelization. Obtained concentration- and time-dependent inter-relationships on HF migration by ‘wound healing’ assays correlate well with previous findings [40,41], wherein the effects of plant extracts on fibroblast migration were established. Similarly, the highest effects in most studies were established after longer incubations, e.g., 24 h [42]. Our study showed that only low concentrations (5 µg/mL) of proanthocyanidins significantly increased HF migration to the ’wound’ bed. In line with these results, the study by Hemmati et al. [43] showed that the lowest tested concentration (2%) of grape seed extract rich in proanthocyanidins improved and accelerated the contraction and closure of wounds, shortening the healing time, while high concentrations (70%) had no promotive effect. This postulates that only small quantities of complex tannins can actively participate in and modulate the wound environment for faster healing. Han et al. [44] reported that low concentrations of proanthocyanidins are optimal for maximal cross-linking of collagen tissue and, thus, increasing the migration of fibroblasts. On the other hand, our obtained fibroblast migration inhibiting effects on high concentrations (20 µg/mL) of PRF can also be favorable in excessive fibroblast activity, which can be detrimental to wound healing, leading to complications, namely hypertrophic scarring, keloid formation, and contractures [45]. Some authors have also suggested that proanthocyanidin-rich extracts may trigger the release of vascular endothelial growth factor [43], elevate the expression of collagen type 1 in fibroblast [42], or enhance ‘wound healing’ by mobilizing the fibroblast in the wound site [10]. Since proanthocyanidins work by multiple mechanisms and are involved in more than one phase of the wound healing process in a positive manner, the PRF could be used as a tool to promote tissue regeneration when incorporated into skin formulations, such as dermal films.

Thin films are receiving attention for drug delivery [46]. Pharmaceutical scientists throughout the world are working on the formulation and development of these dermal systems [24]. Ideal dermal films should have sufficient drug loading capacities, fast dissolution rates, long residence times at the sites of administration, adequate flexibility, thickness, moisture, stickiness, and acceptable physicochemical stability [24,46]. An additional important implication is the selection of polymers and plasticizers, which are defined as the backbone of dermal films and should be safe, non-irritant, and non-toxic [24,25]. Our study analyzed the impacts of polymers MC, HEC, and plasticizer PEG 400 on film thickness, moisture, stickiness, and release of the active substance. Methylcellulose and hydroxyethyl cellulose are excellent, non-toxic, and non-allergenic polymers used as film forming agents and drug carriers that produce moderate strength and good flexibility in thin films with less water vapor barriers due to their hydrophilic nature, which aids in water retention [47,48]. Additionally, polyethylene glycol as a plasticizer is commonly used to impart flexibility, reduce brittles, and enhance other mechanical properties of polymeric films [49]. However, to achieve these effects, the composition and ratio between different polymers, as well as plasticizers, have to be optimized [50].

Panchal et al. [51] pointed out that the amount of plasticizer is critical for film formation and separation properties. Our results indicated that as the concentration of PEG 400 increases, the thickness and stickiness of polymeric film increase as well, while the moisture content in it decreases. Lower moisture can decrease the bulkiness and risk of microbial attack [52], while too high of a film thickness may lead to decreased hardness and increased peeling degree of the film [53]. In other studies [25,54,55], polyethylene glycol appeared to have a direct relationship with adhesiveness, hardness, elasticity, and tensile strength. Contents of polymer MC and HEC were found to be more related to release and diffusion processes. Since most proanthocyanidins are water-soluble polyphenolics [56], the determination of their release properties is of great significance. Our obtained negative correlation between HEC content in films and the released amount of proanthocyanidins was partly in line with the study [25], wherein the authors concluded that HEC concentration should not exceed 2% for optimal release of active substances from HEC-based matrix films. Even though HEC is highly hydrophilic, and hydroxyethyl groups attached to the anhydroglucose units by ether linkages decrease the crystallinity of the active substance (increasing solubility in water) [57], it has sustained release and limited swelling properties [58,59]. The release mechanisms from swellable hydrophilic polymers, such as HEC or MC, are determined by the acceptor medium penetration throughout polymer chains, relaxation degree of hydrated polymers, diffusion of the active substance within swollen media, and possible erosion. When the hydrated viscous layer is formed around the polymers, it may act as a barrier, provoking water penetration from dissolving active substances [55,60].

In general, our selected composition of the polymeric film with the highest ratio between the polymer mixture and PEG 400 (1:8.6), when the polymer mixture was predominated by MC, provided acceptable moisture, stickiness, and 56.5% practical release of proanthocyanidins after 4 h and, therefore, could be regarded as a promising tool for PRF delivery. This and previous reports [10,61,62] indicate that topical application of proanthocyanidins may improve wound healing and histological reorganization of the injured tissues. Nevertheless, there are further concerns that have to be addressed, such as the penetration of proanthocyanidins into the skin, excessive hydrophilicity, high reactivity, safety, chemical instability, and ionizability [63,64,65]. Dermal films must be designed without forgetting that skin permeation is a key factor for effective formulation [64]. This may constitute the object of future studies.

## 4. Materials and Methods

### 4.1. Plant Material

The proanthocyanidin-rich fraction was obtained from the lingonberry (*Vaccinium vitis-idaea* L.) leaves using column chromatography as described previously [29]. Lingonberry leaves for purification were collected from different natural sites in September 2019 in northeast Lithuania and then pooled. Briefly, a crude dry extract of lingonberry leaves was prepared by ultrasonic extraction using air-dried grounded leaves and 80% acetone (the sample/solvent ratio of 1:25), followed by vacuum evaporating and freeze-drying. The obtained light brown powder was dissolved in 50% methanol (1:40), applied to a glass column pre-loaded with Sephadex LH-20 (GE Healthcare Biosciences, Uppsala, Sweden), and washed with two columns of water to remove non-phenolic lingonberry constituents, phenolic acids, arbutin, and four columns of 50% ethanol to elute flavonols. The bound fraction, which was enriched in proanthocyanidins, was eluted with two volumes of 70% acetone. PRF was freeze-dried to obtain the yellowish powder, analyzed, and incorporated into experimental films.

### 4.2. Chemicals and Solvents

Analytical and chromatographic grade solvents were used for this study: acetonitrile, methanol, acetone, acetic acid, hydrochloric, and trifluoroacetic acid from Sigma-Aldrich (Steinheim, Germany), ethanol from Vilniaus degtine (Vilnius, Lithuania). Ultrapure water was obtained by a Milli-Q water purification system from Millipore (Bedford, MA, USA).

Standards used in the phytochemical analysis: procyanidins A1, A2, A4, B1, B2, C1, (+)-catechin, and (−)-epicatechin from Sigma-Aldrich; procyanidin B3 from Extrasynthese (Genay, France).

The following reagents were used: 4-dimethylaminocinnamaldehyde (DMAC), 3-(4,5-dimethylthiazol-2-yl)-2,5-diphenyltetrazolium bromide (MTT) from Sigma-Aldrich, and phosphate-buffered saline (PBS) from Gibco (Carlsbad, CA, USA).

Methylcellulose (viscosity 15 cPs) was purchased from Alfa Aesar (Kandel, Germany). Hydroxyethyl cellulose (average M = ~90 kDa) was purchased from Sigma-Aldrich. Polyethylene glycol 400 (PEG 400) was purchased from Carl Roth (Karlsruhe, Germany).

### 4.3. Cell Culture

The human foreskin fibroblasts (HF) CRL-4001 were originally obtained from the American Type Culture Collection (ATCC, Manassas, VA, USA) and kindly provided by Prof. Helder Santos (University of Helsinki, Finland). HF were cultured in Dulbecco’s Modified Eagle’s GlutaMAX medium (Gibco), supplemented with 1% of antibiotics (10,000 U/mL penicillin, 10 mg/mL streptomycin (Gibco)), and 10% fetal bovine serum (Gibco). Cell cultures were grown 25 cm^2^ falcons at 37 °C in a humidified atmosphere containing 5% CO_2_, and were used until the passage of 10.

### 4.4. Phytochemical Analysis

#### 4.4.1. HPLC-PDA Method

Screening of individual proanthocyanidins and other tannins in the obtained fraction from lingonberry leaves was performed with HPLC-PDA (Waters e2695 Alliance system, Waters, Milford, MA, USA) system using ACE Super C18 (250 mm × 4.6 mm, 3 µm) column (ACT, Aberdeen, UK) and gradient elution consisting of 0.1% trifluoroacetic acid (eluent A) and acetonitrile (eluent B) [29]. Elution formed as follows: 0 min, 90% A; 0–40 min, 70% A; 40–60 min, 30% A; 60–64 min, 10% A; 64–70 min, 90% A at a flow rate of 0.5 mL/min. The injection volume of the sample was 10 µL, and the column temperature was maintained at 35 °C. The identification was made by comparison of retention times and spectra with those of commercially available proanthocyanidins.

#### 4.4.2. DMAC Method

The total proanthocyanidin content in the PRF (before incorporating) and polymeric films (during in vitro release test) was evaluated using a DMAC assay with slight modifications [35]. Three milliliters of DMAC reagent (0.1% in acidified ethanol) were mixed with 20 μL of PRF or samples from in vitro release test and kept for 15 min. The absorbance was measured at 640 nm wavelength using a spectrophotometer (Spectronic CamSpec M550, Garforth, UK), when the reference solution was—3 mL of DMAC and 20 μL of distilled water. The total proanthocyanidin content is expressed as milligrams of procyanidin A2 equivalents per gram of dry weight of fraction (mg A2/g DW).

### 4.5. ‘Wound Healing’ Assay

The PRF effect on human fibroblast migration was assessed using the ‘wound healing’ assay, as described elsewhere [66]. After trypsinization, HF cells were seeded in 24-well plates at a density of 4 × 10^4^ cells/well and incubated for 48 h at 37 °C in a humidified atmosphere containing 5% CO_2_. Then the scratch was made using a 100 µL pipette tip. The cells were washed once with PBS, and the fresh medium containing different concentrations of PRF was added. The final extract concentrations were: 20, 10, and 5 µg/mL. For the selection of these PRF concentrations, the effect on HF cells was performed using a standard MTT assay, as described elsewhere [29]. The wells with the medium (without PRF) were used as a negative control. Each biological repetition contained three replicates. The cells were incubated at 37 °C in a humidified atmosphere containing 5% CO_2_.

‘Wounds’ were captured at intervals of 0, 5, and 20 h from scratch under phase-contrast microscopy at a 4× magnification, in two different areas in each well. The ’wound’ area was analyzed using ImageJ program, version 1.530 (National Institutes of Health, USA).

### 4.6. Development of Experimental Film Composition

#### 4.6.1. Experimental Design and Optimization of the Film Composition

The surface response center composition model (*α* = 1) as an experimental design (Design-Expert 13 (Stat-Ease, Inc., USA)) was applied to model the composition of the film [67,68]. The limits for each component were given: 0.30–0.35 g of MC, 0.05–0.10 g of HEC, and 2.0–3.0 g of PEG 400 (plasticizer). Within the specified constituent material limits, 15 film compositions were generated (Table 3).

The experimental polymeric films were evaluated according to the following parameters: thickness, moisture, stickiness, and release of biologically active compounds. Statistical analyses of the data were performed to evaluate the results of the parameters. The following statistically significant mathematical models were obtained:

1. Linear model (*p* = 0.0001) of the moisture: Y_1_ = −0.973X_1_ − 5.360X_2_ − 2.131X_3_ + 11.208;
2. Linear model (*p* = <0.0001) of the stickiness: Y_2_ = −1.803X_1_ + 0.110X_2_ + 0.171X_3_ + 0.454;
3. Linear model (*p* = 0.0034) of the release of biologically active compounds after 15 min: Y_3_ = −5.718X_1_ − 19.902X_2_ − 0.044X_3_ + 5.445;

Here: Y_1_—the moisture (%) in the polymeric films, Y_2_—the stickiness (N) of the polymeric films, Y_3_—the release (%) of biologically active compounds from the polymeric films after 15 min, X_1_—the amount (g) of methylcellulose, X_2_—the amount (g) of hydroxyethyl cellulose, and X_3_—the amount (g) of polyethylene glycol 400.

In order to obtain the optimal composition of the polymeric films, the following criteria were chosen and arranged in descending order of importance (indicating the number of pluses):

1. Maximum stickiness (5+);
2. Maximum release after 15 min (4+);
3. Minimum moisture (3+).

#### 4.6.2. The Production of the Experimental Polymeric Films

An appropriate amount (Table 3) of methylcellulose, hydroxyethyl cellulose, and 20 g of an aqueous solution of PRF (0.1% solution containing approximately 7.5 mg total of proanthocyanidins based on the DMAC method) were weighed into a Petri dish (50.24 cm^2^) [69,70]. This mixture was stirred on a hotplate stirrer (WiseStir MSH-20D, Wertheim, Germany) until the polymers dissolved. The appropriate amount (Table 3) of plasticizer PEG 400 was then weighed into a Petri dish with an aqueous polymer solution and mixed. The Petri dish with the prepared solution was placed into a shaking incubator (GFL 3032, Burgwedel, Germany), maintaining the temperature at 40 °C, and was kept until constant film weight. The formed film (50.24 cm^2^) was weighed and further analyzed.

#### 4.6.3. Thickness Measurement

The thickness of the experimental films (1.77 cm^2^) was measured (*n* = 3) using a digital micrometer (BGS technic 8427, Wermelskirchen, Germany) and expressed in µm.

#### 4.6.4. Moisture Measurement

The moisture content of the experimental films (1.77 cm^2^) was measured (*n* = 3) using an electronic moisture analyzer (Kern MLS 50-3 HA 160, Balingen-Frommern, Germany) and expressed in %.

#### 4.6.5. Stickiness Measurement

The stickiness of the experimental films (50.24 cm^2^) was measured at several random locations (*n* = 4) using a texture analyzer (TA.XT plus, Godalming, UK) with a mucoadhesion rig (A/MUC) and expressed in N. Conditions for the texture analysis:

(1) The pre-test speed was 1 mm/s.
(2) The test speed was 0.5 mm/s.
(3) The post-test speed was 1 mm/s.
(4) The applied force was 1 N.
(5) The return distance was 10 mm.
(6) The contact time was 10 s.

#### 4.6.6. In Vitro Release Test

The experimental films (1.77 cm^2^, 0.099–0.187 g) were placed in Eppendorf^®^ centrifuge tubes (2 mL) and filled with 2 mL of purified water. The tubes were stored at 32 °C (normal skin surface temperature). Samples were taken after 0.25, 0.5, 0.75, 1, 2, 3, and 4 h by adding the same volume of fresh acceptor medium. Release samples were analyzed spectrophotometrically (Section 4.4.2). The amount of PRF released was calculated from the determined concentration of PRF in the samples and expressed in % and flux (µg/cm^2^).

### 4.7. Statistical Analysis

The results of our studies are presented as mean ± standard deviation. Statistically significant differences were found using one-way ANOVA when the post hoc criterion was Tukey HSD. The correlation was assessed by Spearman’s rank correlation coefficient. The significance level was *α* = 0.05.

## 5. Conclusions

The phytochemical profile and wound-healing properties of PRF from lingonberry leaves were evaluated and an optimal dermal film as a drug delivery system was developed. The considerable richness of proanthocyanidins was confirmed by HPLC-PDA and DMCA methods, while the ‘wound healing’ assay suggested that low concentrations of lingonberry proanthocyanidins may increase human fibroblast migration to the ‘wound‘ bed. Fifteen compositions containing PRF and different concentrations of HEC, MC, and PEG 400 were formulated in order to develop dermal film for the possible wound-healing effect. The experimental design showed that the most appropriate composition was obtained with 0.30 g of MC, 0.05 g of HEC, and 3.0 g of PEG 400 (No. 14) in terms of quality parameters, such as thickness, moisture, stickiness, and ability to release proanthocyanidins. The above results provide firm evidence that topical application of lingonberry proanthocyanidin in the form of an MC-HEC film represents a feasible approach to support dermal wound healing. Future research could continue to elucidate the exact mechanisms of lingonberry proanthocyanidins in wound healing and assess the suitability of the developed polymeric films.

## Figures and Tables

**Table 1:** The parameters of the physical and mechanical properties of the experimental polymeric films.

Films No.	Code *	Thickness (µm)	Moisture (%)	Stickiness (N)
1	MH0.425-P2.5	470 ± 16	4.6 ± 0.4	0.275 ± 0.008
2	MH0.400-P2.5	348 ± 9	4.5 ± 0.2	0.362 ± 0.011
3	MH0.400-P3.0	323 ± 9	4.6 ± 0.9	0.419 ± 0.012
4	MH0.450-P3.0	408 ± 5	3.9 ± 0.3	0.366 ± 0.007
5	MH0.400-P3.0	429 ± 20	4.1 ± 0.4	0.386 ± 0.024
6	MH0.400-P2.0	285 ± 13	7.1 ± 1.0	0.143 ± 0.017
7	MH0.400-P3.0	441 ± 19	4.3 ± 0.1	0.327 ± 0.017
8	MH0.375-P2.5	426 ± 11	5.5 ± 0.4	0.370 ± 0.031
9	MH0.425-P2.5	522 ± 33	5.1 ± 0.4	0.275 ± 0.015
10	MH0.400-P2.0	304 ± 6	5.7 ± 0.5	0.264 ± 0.006
11	MH0.400-P2.0	322 ± 5	6.4 ± 0.8	0.191 ± 0.008
12	MH0.450-P2.0	340 ± 7	6.3 ± 0.3	0.179 ± 0.017
13	MH0.350-P2.0	262 ± 18	6.3 ± 0.7	0.276 ± 0.010
14	MH0.350-P3.0	373 ± 19	4.4 ± 0.3	0.412 ± 0.027
15	MH0.375-P2.5	369 ± 25	4.8 ± 0.2	0.319 ± 0.005

Note: *—MH is a mixture of polymers (MC, HEC), the number next to it indicates the total amount (g), P is PEG 400, and the number next to it indicates its amount (g).

**Table 2:** Theoretical and experimental values of the selected polymeric film properties.

Properties	Stickiness (N)	Release after 0.25 h (%)	Moisture (%)
Theoretical value	0.432	2.60	4.3
Experimental value	0.412	3.12	4.4

**Table 3:** The compositions of the experimental polymeric films.

Films No.	MC Amount (g)	HEC Amount (g)	PEG 400 Amount (g)	The Ratio between MC:HEC	The Ratio between Polymers Mixture: PEG 400	Code *
1	0.350	0.075	2.5	4.7:1	1:5.9	MH0.425-P2.5
2	0.325	0.075	2.5	4.3:1	1:6.3	MH0.400-P2.5
3	0.300	0.100	3.0	3.0:1	1:7.5	MH0.400-P3.0
4	0.350	0.100	3.0	3.5:1	1:6.7	MH0.450-P3.0
5	0.325	0.075	3.0	4.3:1	1:7.5	MH0.400-P3.0
6	0.350	0.050	2.0	7.0:1	1:5.0	MH0.400-P2.0
7	0.350	0.050	3.0	7.0:1	1:7.5	MH0.400-P3.0
8	0.300	0.075	2.5	4.0:1	1:6.7	MH0.375-P2.5
9	0.325	0.100	2.5	3.3:1	1:5.9	MH0.425-P2.5
10	0.300	0.100	2.0	3.0:1	1:5.0	MH0.400-P2.0
11	0.325	0.075	2.0	4.3:1	1:5.0	MH0.400-P2.0
12	0.350	0.100	2.0	3.5:1	1:4.4	MH0.450-P2.0
13	0.300	0.050	2.0	6.0:1	1:5.7	MH0.350-P2.0
14	0.300	0.050	3.0	6.0:1	1:8.6	MH0.350-P3.0
15	0.325	0.050	2.5	6.5:1	1:6.7	MH0.375-P2.5

Note: *—MH is a mixture of polymers (MC, HEC), the number next to it indicates their total amount (g), P is PEG 400, and the number next to it indicates its amount (g).

## Data Availability

All data generated during this study are included in this article.
